# Bioluminescent, Nonlytic, Real-Time Cell Viability Assay and Use in Inhibitor Screening

**DOI:** 10.1089/adt.2015.669

**Published:** 2015-10-01

**Authors:** Sarah J. Duellman, Wenhui Zhou, Poncho Meisenheimer, Gediminas Vidugiris, James J. Cali, Prson Gautam, Krister Wennerberg, Jolanta Vidugiriene

**Affiliations:** ^1^ Research and Development, Promega Corporation, Madison, Wisconsin.; ^2^ Promega Biosciences, LLC, San Luis Obispo, California.; ^3^ Institute for Molecular Medicine Finland, University of Helsinki, Helsinki, Finland.

## Abstract

*Real-time continuous monitoring of cellular processes offers distinct advantages over traditional endpoint assays. A comprehensive representation of the changes occurring in live cells over the entire length of an experiment provides information about the biological status of the cell and informs decisions about the timing of treatments or the use of other functional endpoint assays. We describe a homogeneous, nonlytic, bioluminescent assay that measures cell viability in real time. This time-dependent measurement allowed us to monitor cell health for 72 h from the same test samples, distinguish differential cell growth, and investigate drug mechanism of action by analyzing time- and dose-dependent drug effects. The real*-*time measurements also allowed us to detect cell death immediately (>75% signal decrease within 15 min of digitonin addition), analyze drug potency versus efficacy, and identify cytostatic versus toxic drug effects. We screened an oncology compound library (Z′ = 0.7) and identified compounds with varying activity at different time points (1.6% of the library showed activity within 3 h, whereas 35.4% showed a response by 47 h). The assay compared well with orthogonal endpoint cell viability assays and additionally provided data at multiple time points and the opportunity to multiplex assays on the same cells. To test the advantage of time-dependent measurements to direct optimal timing of downstream applications, we used the real-time cell viability assay to determine the ideal time to measure caspase activity by monitoring the onset of cell death and multiplexing a luminescent caspase activation assay on the same test samples.*

## Introduction

Cell viability is a universal endpoint that is measured in many drug discovery efforts across a variety of disease models. There are numerous cell viability assays available that measure different biomarkers, including adenosine triphosphate (ATP), reducing potential, and cellular enzyme function. Each biomarker provides advantages based on the experimental goals, but most assays are used in endpoint formats that destroy the cells and are incompatible with the kinetic or real-time analysis of compound toxicity. This format poses a challenge because toxicity is both dose- and time-dependent. For a given dose range, the onset and progression of toxicity are observed at different time points for different compounds. A better approach would enable continuous reads that reveal the emergence of dose-dependent toxicity over time for a given set of samples. Otherwise, each endpoint requires a separate set of samples.

One strategy for real-time monitoring of cell viability examines electrical impedance.^1,2^ This approach has the advantage of being label free, so reagents orthogonal to the experiment parameters are not required. However, impedance measurements require a special reader and special assay plates. Furthermore, the technique does not work well with suspension cells and can overestimate cell proliferation in senescence while underestimating cell proliferation in cell lines that tend to aggregate. A more universal and readily accessible method is needed.

We describe a newly developed bioluminescent assay that measures cell viability in real time through a simple, add-and-read format, using a standard luminometer. The assay detects cellular reducing capacity, which is a classic marker of cell viability. Two assay components are required, a purified luciferase enzyme^3^ and a cell viability probe, that is, a luciferase prosubstrate. Both components are added to cell cultures, where viable cells reduce the probe to a form that is active with the luciferase present in the medium. Since the reduced probe is rapidly utilized by luciferase, it does not accumulate. Rather, a steady-state signal is maintained that correlates to the number of viable cells present at a given moment in time; as cells divide and expand, the signal increases; as cells die, the signal decreases. Because the assay reagents are nontoxic to cells and the turnover of the prosubstrate is slow, continuous reads can be obtained over extended time frames (*e.g.*, 72 h). In contrast, conventional reducing potential assays monitor the cellular conversion of resazurin or a tetrazolium salt to a stable product that accumulates over time in a process that kills the cells.^4–8^ While these assays provide adequate snapshots of cell viability at a given endpoint, they cannot provide kinetic data for a given sample or sample set.

To test the value of this new assay for screening in a dose–response mode, we applied a 308-compound oncology library to cultured cells. The assay allowed us to characterize the time-dependent potencies of library compounds in a facile and cost-effective way.

We were also able to use the time-dependent cell viability measurements to predict the optimal timing of a downstream measurement of apoptosis. Because the assay was well tolerated by cells and the assay chemistry is compatible with many different multiplexes, including other luminescent assays without the need for spectral filters, we were able to establish the optimal time to multiplex a luminescent apoptosis assay to capture the caspase activation window and avoid false-negative results. This multiplex provided more meaningful data through interrogation of the same experimental samples, thereby providing an internally controlled dataset.

## Materials and Methods

### Materials

All cell lines were obtained from ATCC. A549 lung carcinoma cells were cultured in F-12K media (ATCC). K562 chronic myelogenous leukemia cells and THP-1 acute monocytic leukemia cells were cultured in RPMI media (Gibco). MDA-MB-231 breast cancer cells were cultured in Dulbecco's modified Eagle's medium (Gibco). All media were supplemented with 10% fetal bovine serum (HyClone) and penicillin/streptomycin (pen/strep; Gibco). RealTime-Glo MT Cell Viability Assay, CellTiter-Glo, CellTiter-Fluor, and Caspase-Glo 3/7 were obtained from Promega Corporation. White 384-well assay plates were purchased from Corning, Inc. All reagents were purchased through Sigma, unless otherwise noted.

### Monitor Cell Growth in Real Time

A549 cells (1,000/well) were plated in a 384-well plate in 40 μL of media. The cells were incubated overnight in a 37°C, 5% CO_2_-humidified atmosphere. The media was removed and replaced with 72 μL of CO_2_-independent media (Gibco) containing real-time cell viability reagents (RealTime-Glo MT Cell Viability Assay). The plate was read on a Tecan M200 plate reader set at 37°C every 30 min for 24 h. At 24 h, 8 μL of 2,000-μg/mL digitonin in CO_2_-independent media was added to the cells, and the luminescence was determined every 15 min for 5 h. The A549 cell line was chosen to represent a commonly used adherent cancer cell line.

Fresh rat hepatocytes were obtained from Bioreclamation IVT and plated in 72-μL InVitroGRO CP Medium containing the RealTime-Glo reagents. The plate was read on a Tecan M200 plate reader with gas control module (GCM) set at 5% CO_2_ and 37°C every 30 min for 24 h. At 24 h, 8 μL of 2,000-μg/mL digitonin in InVitroGRO CP Medium was added to the cells, and the luminescence was determined every 15 min for 5 h. Hepatocytes were chosen to represent a commonly used nonproliferating primary cell line.

### Monitor Drug Treatment

A549 cells (500/well) were plated in 40-μL media containing 2 × RealTime-Glo reagents. A thapsigargin titration was prepared in media at 2 × concentrations and added to the plate at an equal volume. K562 cells (500/well) were plated in 40-μL media containing 2 × RealTime-Glo reagents. A panobinostat titration was prepared in media at 2 × concentrations and added to the plate at an equal volume. Luminescence was read every hour for 72 h on the Tecan M200 GCM (37°C/5% CO_2_). Half-maximal effective concentration (EC_50_) values were calculated using GraphPad Prism® software, version 6.03. A549 and K562 cells were chosen to include a variety of cell types in our analysis and also to test the utility of this assay in both adherent and suspension cells.

### Oncology Small-Molecule Library Screening

The Institute for Molecular Medicine Finland (FIMM) compound collection consists of 308 active compounds, including FDA/EMA-approved anticancer drugs and emerging investigational and preclinical compounds, covering a wide range of molecular targets. The compounds were obtained from the National Cancer Institute Drug Testing Program (NCI DTP) and the following commercial chemical vendors: Active BioChem, Axon Medchem, Cayman Chemical Company, ChemieTek, Enzo Life Sciences, LC Laboratories, Merck, Santa Cruz Biotechnology, Selleck, Sequoia Research Products, SGC, Sigma-Aldrich, and Tocris Biosciences. To ensure that compounds were soluble and at the intended concentration, each compound was dissolved within reported solubility ranges and some compounds were dissolved in an aqueous solution because of poor solubility in dimethyl sulfoxide (DMSO). All compound stocks were also visually inspected for precipitation at the time of dissolving and at any time they were handled. The potency of library compounds was tested to ensure that it matches the potency described in the literature, and a set of control cell lines was screened every 2 months to ensure that potencies stayed constant over time.

The compounds were either dissolved in 100% DMSO or Milli-Q water and dispensed in tissue culture-treated 384-well plates (Corning, Inc.) using an acoustic liquid handling device, Echo 550 (Labcyte, Inc.). The compounds were plated at five different concentrations in 10-fold dilutions covering a 10,000-fold concentration range (*e.g.*, 1–10,000 nM). The compounds were dispensed in 2.5- or 25-nL volume per well to keep the final DMSO concentration no higher than 0.1% in the cell culture medium. As the negative and positive control, 100% DMSO and 100-mM benzethonium chloride (BzCl), respectively, were added to the wells to give final concentrations of 0.1% DMSO and 100-μM benzethonium chloride. In addition, to confirm no effect of DMSO on cell culture or readout assay chemistries, no DMSO control samples with or without cells were tested and showed no significant difference compared to negative and positive controls used in the screen. In separate experiments, these cell viability assays were tested to determine the effect of DMSO, and the presence of DMSO in the samples up to 1% had no effect on the performance of these assays (data not shown).

The library was screened with K562 cells. This cell line represents a commonly used cancer cell type that is grown in suspension. To the predispensed compounds, 5 μL of RPMI media was added. K562 cells (1,500/well) were plated in 20-μL RPMI media containing 1 × RealTime-Glo MT Cell Viability Assay reagents. Luminescence was read on a Tecan Infinite M200 plate reader at 3, 6, 12, 22, 31, and 47 h. The protocol for the screen performed using the real-time cell viability assay is described in *Table 1*. Two separate sets of the library were used for two independent screens to compare the reproducibility of the RealTime-Glo MT Cell Viability Assay. All the reagents were dispensed using Multidrop Combi (Thermo Scientific). The robustness of the assay was determined by calculating the signal-to-background (S/B) (average signal from vehicle control cells/average signal from 100-μM benzethonium chloride-treated cells), signal-to-noise (S/N) ([average signal from vehicle control cells/average signal from 100-μM benzethonium chloride-treated cells]/standard deviation from 100-μM benzethonium chloride-treated cells), and Z′ value.^9^ Z′ was calculated using the values from the vehicle control cells (signal) and 100-μM benzethonium chloride-treated cells (inhibited).

**Table 1.** Oncology Small-Molecule Library Screen Using the Real-Time Cell Viability Assay

Step	Parameter	Value	Description
1	Library compounds	2.5 or 25 nL	10-fold dilutions, five concentrations
2	Controls	25 nL	100% dimethyl sulfoxide, 100-mM benzethonium chloride
3	Compound diluent	5 μL	RPMI complete media
4	Cells and reagent	20 μL	1,500 K562 cells/well, cell suspension containing 1 × RealTime-Glo MT Cell Viability Assay reagents
5	Incubation	Various times	37°C, 5% CO_2_
6	Real-time assay readout	Luminescence	Measure at 3, 6, 12, 22, 31, and 47 h from the same assay wells at each read

**Step Note**

1. Tissue culture-treated 384-well plate.

Two additional screens were performed for comparison to the RealTime-Glo screens. These screens were performed identically to the RealTime-Glo screen, and cell viability was measured at 47 h. In brief, for each screen, 5 μL of RPMI media was added to the predispensed compounds. K562 cells (1,500/well) were plated in 20-μL RPMI media. ATP levels were measured using the CellTiter-Glo Assay (Promega). Live cell protease was measured using the CellTiter-Fluor Assay (Promega). Fluorescence and luminescence measurements were performed using a Tecan Infinite M200 plate reader.

### Data Analysis and Scoring of Drug Sensitivity

The data obtained from the plate readers were uploaded and analyzed in Dotmatics Browser/Studies software (Dotmatics Ltd.), as described previously.^10,11^ For data analysis, we have used a methodology that has been described elsewhere and is freely available.^11^ The method integrates a multiparameter analysis, including the potency (EC_50_), slope of the dose–response curve, the area under the curve, and the maximum effect of the drug, into a single metric termed the drug sensitivity score (DSS). The DSS R-package and its source code are freely available at https://dss-calculation.googlecode.com/svn/trunk/. Based on their DSS, compounds were divided into five groups: inactive (DSS = 0), low active (DSS > 0–5), semiactive (DSS > 5–10), active (DSS > 10–20), and very active (DSS > 20). To evaluate the effect of library compounds on cell viability, the DSS was calculated for each compound. Data from one representative screen of the RealTime-Glo MT Cell Viability Assay are summarized in *Supplementary Table S1* (Supplementary Data are available online at www.liebertpub.com/adt). Data from the screens that measured ATP levels and an orthogonal cell viability assay (CellTiter-Fluor) are summarized in *Supplementary Tables S2* and *S3*, respectively. Compounds with DSS < 5 had no or very slight effect on measured responses and are not included in the tables.

To determine compounds that showed a differential response between each assay, we calculated the selective DSS (sDSS). sDSS was calculated by subtracting the average cell viability DSS of one assay from the average cell viability DSS of the comparison assay. To determine the cutoff for compounds with a differential response, we calculated the sDSS values that were three standard deviations above and below the average sDSS of all compounds within each comparison.

### Multiplex with Caspase Assay

THP-1 cells (2,000/well) were plated in media containing 2 × RealTime-Glo reagents. THP-1 cells were used to show utility with another cell type. Terfenadine and doxorubicin were prepared as 2 × solutions in cell culture media and added to the assay plate in an equal volume for final concentrations of 20-μM terfenadine or 1-μM doxorubicin. Luminescence was monitored every 4 h on a Tecan M200 GCM (37°C/5% CO_2_). At various time points, the Caspase-Glo 3/7 assay was prepared containing 200 μM of a NanoLuc inhibitor and added to the test samples at an equal volume. The luminescence signal was determined from the caspase assay 1 h after the caspase detection reagent was added. The NanoLuc inhibitor was used because the Caspase-Glo 3/7 assay contains a high level of reducing agent that may contribute to an increased background signal from the RealTime-Glo assay. We have found that if the Caspase-Glo 3/7 signal is read within 1 h of reagent addition or the media is removed before the addition of caspase detection reagent, the NanoLuc inhibitor is often not required.

## Results

### Assay Principle and Real-Time Applications

**Fig. 1.**
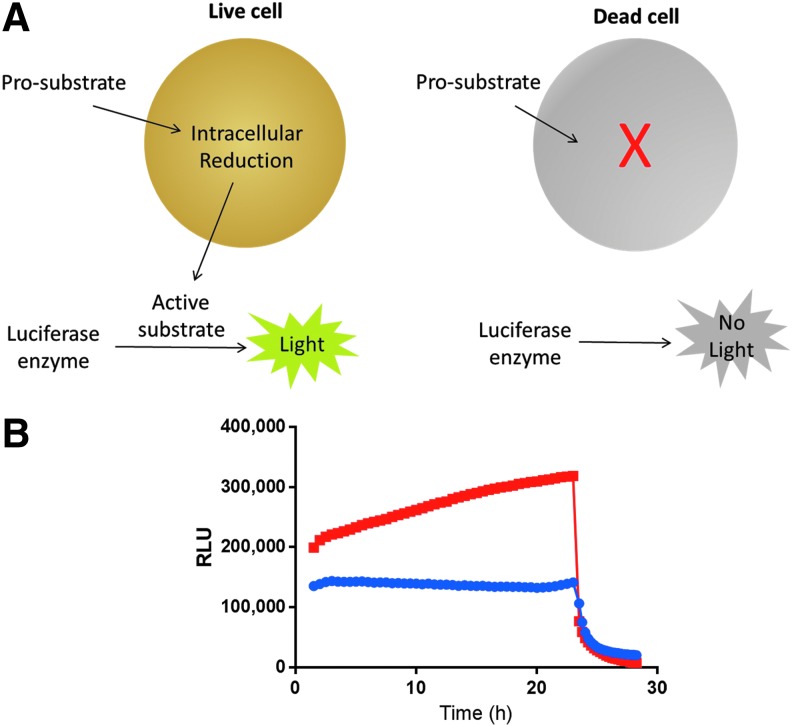
Real-time cell viability assay. **(A)** Real-time cell viability assay overview. **(B)** Cell viability signals were monitored in proliferating A549 lung cancer cells (*red*) or in nonproliferating primary rat hepatocytes (*blue*) every 30 min for 24 h. After the 24-h reading, 200-μg/mL digitonin was added to the cells and signal monitoring was resumed at 15-min intervals.

The real-time cell viability assay consists of two components, a luciferase enzyme and a prosubstrate (*Fig. 1A*), which are added to the cell culture media. The prosubstrate is internalized by the cells, whereas the luciferase enzyme remains in the media. The prosubstrate is reduced intracellularly to form the active luciferase substrate. The active substrate exits the cell and is used rapidly by the luciferase enzyme. The active substrate does not accumulate, which allows an immediate detection of cell death. When the cells die, they no longer reduce the prosubstrate and the constant flow of active substrate is stopped. Any active substrate already generated is rapidly used by the luciferase enzyme. Both the prosubstrate and the luciferase enzyme are stable in cell culture conditions for >96 h (data not shown). The reaction between the luciferase and the substrate is very bright, which allows low usage of prosubstrate and low product buildup in live cells. The assay reagents are well tolerated by cells (*Supplementary Fig. S1*), likely due to the low concentration of substrate that is generated at any given time.

As the signal corresponds to the number of viable cells in culture, we set out to determine if the assay can distinguish between dividing and nondividing cells in culture. We plated either proliferating A549 lung cancer cells or nonproliferating primary rat hepatocytes (*Fig. 1B*). The real-time cell viability assay was added to the cell culture media, and luminescence was monitored every 30 min for 24 h. The proliferating cancer cell line showed increasing signal over 24 h, as expected, since the cell number would increase during the time course. In contrast, the nonproliferating primary cell line showed a constant signal over the time course, as expected, from viable cells that are not dividing. To determine whether we could detect cell death immediately, we added digitonin, which rapidly permeabilizes the cell membrane. The first read was performed 15 min after the addition of digitonin, and both cell lines showed an immediate decrease in luminescent signal.

### Time- and Dose-Dependent Drug Effects

To determine if the real-time cell viability assay could be used in kinetic mode to detect changes in EC_50_, we analyzed the effect on cell viability of small-molecule drugs over time. A549 cells were treated with thapsigargin and K562 cells were treated with panobinostat, both in media containing the real-time cell viability assay reagents. The luminescence signal was determined every hour for 72 h from the same assay wells. By watching the cell viability signals over time, we were able to pinpoint the timing of drug-induced cytotoxicity.

**Fig. 2.**
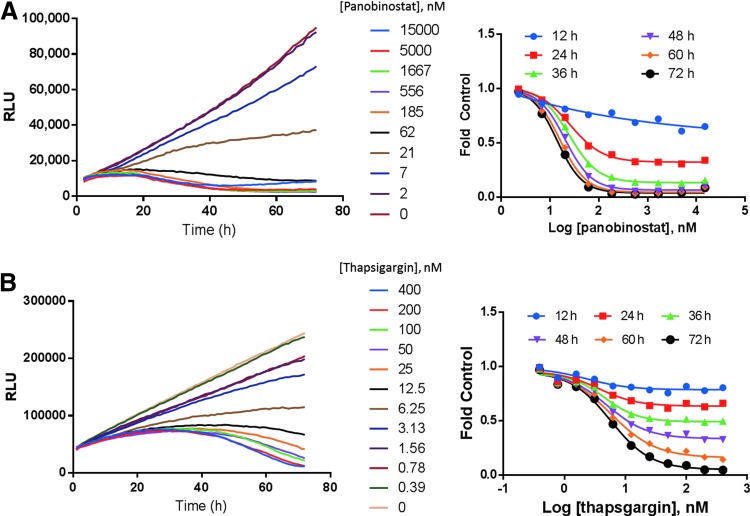
Time- and dose-dependent drug effects. Cells were plated in medium containing the real-time cell viability reagents and treated with compounds. **(A)** K562 cells were treated with panobinostat and **(B)** A549 cells were treated with thapsigargin. Luminescence was monitored every hour for 72 h. The fold change in cell viability (fold control, drug-treated cells/vehicle control cells) at each concentration was determined.

Panobinostat and thapsigargin (*Fig. 2*) killed cells more slowly than digitonin (*Fig. 1B*). Signals from untreated control cells showed increasing signals over time, whereas signals from drug-treated cells decreased with increasing drug concentrations. In addition to the timing of the drug effect, we were also able to perform a detailed interrogation of the various drug doses. In the thapsigargin example, the 6.25-nM dose showed a static cell growth profile (*Fig. 2B*), whereas the panobinostat data show a similar effect around 21 nM (*Fig. 2A*).

The real-time cell viability assay enabled a convenient approach for comparing drug efficacy and potency. We determined the EC_50_ value every 12 h over a 72-h time course (*Table 2*). Panobinostat reached the maximum efficacy by 48 h with 90% inhibition and no further decrease in cell viability at later time points. However, panobinostat potency continued to increase after 48 h as the EC_50_ trended lower. In contrast, thapsigargin showed a potent response as early as 24 h, and this potency did not change through 72 h, as seen by no significant change in the EC_50_ values. The efficacy, however, changed throughout the time course because the maximum effect increased from 19% inhibition at 12 h to 95% inhibition at 72 h.

**Table 2.** Time-Dependent Changes in Potency and Efficacy

	EC_50_ (nM) and maximum percentage inhibition					
Measurement	12 h	24 h	36 h	48 h	60 h	72 h
Panobinostat EC_50_^a^	ND	25.6	24.9	20.6	16.2	14.1
Panobinostat maximum percentage inhibition^a^	35	66	85	89	91	91
Thapsigargin EC_50_^b^	ND	3.7	4.6	5.0	5.8	5.7
Thapsigargin maximum percentage inhibition^b^	19	34	50	67	86	95

^a^ Panobinostat experiments were performed with K562 cells.

^b^ Thapsigargin experiments were performed with A549 cells.

EC_50_, half-maximal effective concentration; ND, not determined.

### Small-Molecule Screening

To test the utility of the real-time cell viability assay for high-throughput screening, we performed a screen using the FIMM oncology library. This library includes 308 biologically active small-molecule drugs, including FDA-approved anticancer compounds and new investigational oncology compounds. Each drug is plated at five concentrations in a 10-fold dilution series. Screening each compound in a dilution series allowed us to determine the approximate activity of each drug with time and compare the results from the real-time cell viability assay to other cell viability assays. The effect of each drug was scored using the DSS, as described elsewhere.^10,11^ In brief, this quantification takes into account not only the slope of the dose–response curve but also the area around the curve and the maximum effect of the drug, thereby providing more information than EC_50_ values alone. An increase in DSS correlates with an increase in maximum effect and a decrease in EC_50_ (*i.e.*, a more active drug).

**Fig. 3.**
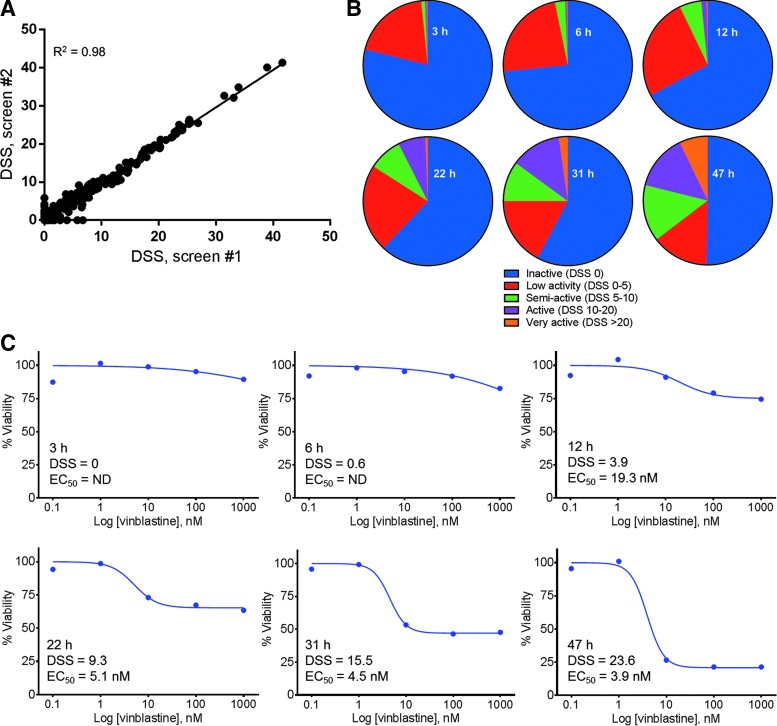
Oncology small-molecule library screening. **(A)** The real-time cell viability assay was screened against the Institute for Molecular Medicine Finland (FIMM) Oncology Library twice to determine the reproducibility of the assay. **(B)** The DSS was calculated at each time point, and drugs were ranked based on their potency. These pie charts represent the proportion of the library with each DSS, including inactive DSS = 0 (*blue*), low activity DSS > 0–5 (*red*), semiactive DSS > 5–10 (*green*), active DSS > 10–20 (*purple*), and very active DSS > 20 (*orange*). **(C)** The effect of vinblastine on cell viability was monitored at each time point, and the DSS and EC_50_ values were calculated. DSS, drug sensitivity score; EC_50_, half-maximal effective concentration; ND, not determined.

The compounds were pre-dispensed in 384-well assay plates and K562 cells were added in media containing the real-time cell viability reagents. The cells were incubated with the drugs for a total of 47 h, and the luminescent signal was read at multiple time points during the incubation. The library was screened against the real-time cell viability assay twice to determine the reproducibility of this assay. The two screens correlated well as shown by the DSS analysis in *Figure 3A*. The assay provided advantages for high-throughput screening, including large assay window (S/B = 17 and 18, S/N = 62 and 66, for screens 1 and 2, respectively), no additional plating steps (reagents added with cell suspension), and robust performance (Z′ = 0.70 and 0.71 for screens 1 and 2, respectively).

By measuring the cell viability signal at each time point, we could monitor the entire library for drugs that acted quickly (*e.g.*, DSS > 5 within 3 h) and slowly (*e.g.*, no activity until later time points) (*Fig. 3B* and *Supplementary Table S1*). We could also monitor drugs that increased in potency throughout the time course. By 47 h, 8% of the library was highly active and just over half of the library showed no activity. *Table 3* shows 10 drugs from the screen with varying activity. YM155,^12^ a survivin inhibitor, was one of the most potent drugs and the strong decrease in cell viability was detected as early as 3 h, suggesting the cells died rapidly upon drug treatment. Many other drugs, such as idarubicin and MEK162, exhibited little or no activity at the earliest time points, but increased in activity as the time course progressed. Vinblastine is another example of a drug that showed no activity at the earliest time points, but increased in activity over the 47-h experiment, ending with a highly active DSS of 23.6 (*Fig. 3C*). The EC_50_ for vinblastine did not change significantly between 22 and 47 h, while the DSS went from 9.3 to 23.6 due to the increase in the maximum effect.

**Table 3.** Library Compounds That Represent Differences in Time-Dependent Cytotoxicity

	DSS						
Compound	3 h	6 h	12 h	22 h	31 h	47 h	Mechanism of action^a^
Bortezomib	0	0	1.4	9.1	16.6	25.3	26S proteasome inhibitor
Idarubicin	0	0	0	2.2	4.7	8.5	Topoisomerase II inhibitor
Indibulin	0	0	0	3.9	8.6	12.8	Destabilizes tubulin polymerization
MEK162	0	0	0	0	2.7	5.9	MEK1/2 inhibitor
Nilotinib	0	0	0.7	11.7	23.7	34.0	Bcr-Abl tyrosine kinase inhibitor
Stattic	5.0	5.0	3.9	4.5	4.7	6.0	Inhibits STAT2 activation/dimerization^b^
Temsirolimus	0	0	0	3.7	7.3	9.5	mTOR serine/threonine kinase inhibitor
Tipifarnib	6.8	7.6	7.0	9.4	11.5	14.6	Inhibits farnesylation of proteins
Vincristine	1.6	2.2	4.8	10.8	17.3	25.6	Inhibits microtubule formation
YM155	23.9	25.8	30.0	31.5	32.0	33.1	Survivin inhibitor

^a^ Mechanisms of action were found using the NCI Drug Dictionary.^16^

^b^ Mechanism of action for Stattic is reported elsewhere.^17^

DSS, drug sensitivity score.

**Fig. 4.**
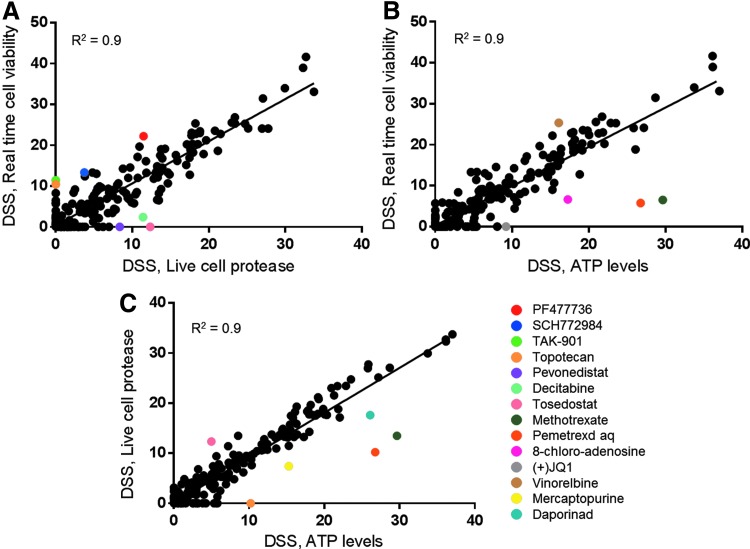
Correlation plots comparing DSS values among the cell viability assays. **(A)** Comparison between the real-time cell viability and live cell protease assays. **(B)** Comparison between the real-time cell viability and adenosine triphosphate (ATP) level assays. **(C)** Comparison between the live cell protease and ATP level assays. Compounds showing a differential response between assays are shown in *color* and labeled with compound name.

To determine how the results from the real-time cell viability assay compared with other cell viability assays, we also performed the screen with two additional assays. These assays measured different biomarkers of cell health, ATP levels and a protease active only in viable cells. Since these assays are endpoint assays, we performed the analysis at the 47-h time point since performing these assays over time would require another full screen of 308 compounds at multiple concentrations for each time point. The ability of the real-time cell viability assay to measure cell viability at multiple time points from one screen is particularly powerful when compared to these endpoint assays that would require us to set up an entirely new screen at each time point. The ATP level assay (S/B = 31.5, S/N = 149, and Z′ = 0.76) and the live cell protease assay (S/B = 6.7, S/N = 242, and Z′ = 0.68) also performed well. All of the assays correlated well suggesting that the real-time cell viability assay can reliably be used to analyze drug activity (*Fig. 4*). Each assay had a few drugs that showed a differential response, which is expected when measuring three different biomarkers of cell viability. For example, methotrexate and pemetrexed are antimetabolites that inhibit purine biosynthesis, which leads to a decrease in ATP levels.^13–15^ As expected, the assay that measured the level of ATP showed a much stronger response to these drugs compared to the other screens. Also, TAK-901 and PF477736 showed stronger responses with the real-time cell viability assay compared to the live cell protease assay. The DSS from the ATP assay is in-between the DSS values from these two assays, suggesting that the metabolic biomarkers of reducing potential and ATP may be more affected by the mechanism of action of these drugs. The percentage of the library in each DSS category was determined at each real-time cell viability time point (*Fig. 3B*) and the 47-h time point with the endpoint assays (*Table 3*). Many drugs increased in potency over the time course, which was easily determined using the real-time cell viability assay. The DSS percentages at 47 h also correlated well with those generated from the other cell viability assays, as summarized in *Table 4*. The detailed analysis of each screen can be found in *Supplementary Tables S1*–*S3*.

**Table 4.** The Percentage of the Small-Molecule Library with Each DSS

		Percentage of compounds with DSS indicated							
		Real-time cell viability						Live cell protease	ATP levels
Category	DSS	3 h	6 h	12 h	22 h	31 h	47 h	47 h	47 h
Inactive	0	78.9	73.4	67.2	62.0	57.8	50.3	50.0	45.5
Low activity	0–5	19.5	23.4	25.6	22.1	17.2	14.3	17.5	20.5
Semiactive	5–10	1.0	2.6	5.5	8.4	10.1	14.3	12.0	13.3
Active	10–20	0.3	0.3	1.3	6.8	12.7	14.0	15.6	14.9
Very active	>20	0.3	0.3	0.3	0.6	2.3	7.1	4.9	5.8

ATP, adenosine triphosphate.

### Drug Activation of Apoptosis

We were interested in determining which small molecules induce cell death through the apoptotic pathway. Apoptosis is often measured by detecting the activation of the caspase proteases. The challenge with this analysis is the transient and short-lived activation of these enzymes. If a caspase activation assay is applied to the cells too early or after the cells are dead and apoptosis is complete, the assay result will be negative, suggesting no caspase activation and therefore no apoptosis. The window of caspase activation may simply have been missed, therefore resulting in a false-negative result. We set out to determine whether we could use the real-time cell viability assay to determine an optimal window of time, in which to multiplex a caspase activation assay to prevent missing the apoptotic window.

**Fig. 5.**
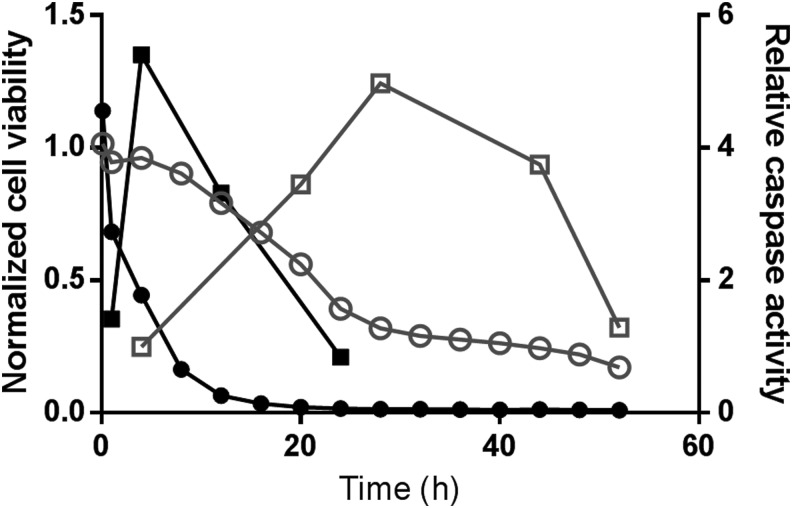
Timing of caspase activation. THP1 cells were grown in media containing the real-time cell viability reagents and treated with 20-μM terfenadine or 1-μM doxorubicin. Cell viability was monitored every 4 h. At various time points, caspase activation was determined. Relative caspase activity and normalized cell viability were calculated by dividing the values from drug-treated samples by the vehicle control values. Doxorubicin treatment: cell viability (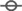), caspase activation (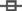). Terfenadine treatment: cell viability (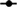), caspase activation (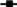).

The real-time cell viability assay was added to cells, and luminescence was monitored every 4 h for 48 h after drug treatment. A caspase activation assay was multiplexed with the real-time cell viability assay at multiple time points throughout the time course (*Fig. 5*). Terfenadine resulted in significant cell death within the first 4 h of treatment. The caspase activation in these cells peaked around 4 h, which corresponds well with the real-time measurement of cell viability. Cell viability was unaffected by doxorubicin at these early time points, and correspondingly, there was no caspase activation within the first 4 h. In contrast, the window of caspase activation induced by doxorubicin began around 20 h, which corresponded with a decrease in cell viability, whereas caspase activation induced by terfenadine was no longer detectable at 24 h. These two drugs show the importance of being able to target the caspase activation window since the timing of apoptosis can differ significantly with different drugs. In both cases, when cell viability reached ∼50% of control cells, the caspase activation window could be detected. As an added benefit, the luminescent caspase assay was multiplexed directly on the wells containing real-time cell viability assay. Because the signal from the cell viability assay immediately decreases when the cells are lysed, a luminescent assay with a lytic component can be multiplexed without the need for spectral filters. The lysis component in the caspase assay killed the cells, which immediately decreased the real-time cell viability signal, and the remaining luminescence at the next read was from the caspase assay.

## Discussion

Innovative technologies that allow drug discovery efforts to become more streamlined, affordable, and informative are needed. We describe a new cell viability assay that allows more detailed analysis of drug effects with time through a standard plate-based luminescence reading. This assay utilizes two components, a luciferase enzyme and prosubstrate, which are added to cell culture media. There is no need for cell engineering and the components can be combined with the cell suspension or drug dosing to avoid additional plating steps.

The real-time cell viability assay allowed us to perform many unique analyses that are currently more laborious, expensive, and inconvenient. This assay correlated well with the number of viable cells in the well as reflected by increasing signals in proliferating cells and static signals in nondividing primary cell lines. The ability to distinguish these growth profiles indicates that the assay could be used to examine cell treatments that lead to differential cell growth and not only cytotoxicity. The assay also detected drug-induced cell death immediately. This temporal analysis of drug effects allowed fast-acting drugs (*e.g.*, digitonin) to easily be distinguished from slow-acting drugs (*e.g.*, thapsigargin). Being able to monitor the drug effect as many times as needed over a desired time course allowed us to examine drug potency versus efficacy, and determine EC_50_ values at any time point.

To compare the cell viability results from the real-time cell viability assay to other established cell viability assay technologies, we set up multiple screens looking at 308 active compounds in dose–response mode. The real-time cell viability assay not only correlated well with the other assays in reporting comparable drug effects but also provided significantly more information by allowing us to easily monitor the drug's effect over time. Each of the endpoint assays would have required us to set up an entirely separate screen at each time point as these assays are lytic or toxic to the cells. Although we did not perform any downstream applications after the real-time cell viability screen was complete, we could have multiplexed a number of assays with the real-time cell viability assay as the assay chemistry is compatible with a variety of assays and the cells are still viable. This opportunity would allow for further conservation of samples and reagents, as well as very reliable comparison data between assays since the readings from the cell viability assay and the multiplexed assay would be made from the same cells in the same well. In this way, the real-time cell viability assay could be used to normalize the signal of other assays (*e.g.*, reporter assays) to cell number in each well, providing an internally controlled dataset.

We decided to use this broad multiplexing compatibility to help us determine when to analyze for apoptosis. When determining mechanism of action of a drug, it is often useful to determine through which death pathway the cells are progressing. Apoptosis can be a difficult measurement to make, however, because the caspase proteases are active for only a short time, and if that window is missed, a false-negative result is possible. We wanted to see whether cell death reported by the real-time cell viability assay would be a useful way to plan the addition of the caspase activation assay. We found that monitoring cell death in real time worked very well in helping us predict when the caspase enzymes were active. In general, a 50% drop in cell viability compared to control cells occurred at a time when the caspase activity was detectable. In addition, we could add the caspase activation assay to the same wells as the real-time cell viability assay, to very conveniently analyze the same cells with both assays and conserve cells and reagents. This multiplexing compatibility increases the potential for multiplexed assays to include many different measurements along with caspase activation (*e.g.*, reporter assays, metabolite levels, cytotoxicity).

The real-time cell viability assay is the first plate-based assay to easily measure cell viability in real time in live cells. It is a nonlytic homogeneous bioluminescent assay that provides extensive multiplexing options, including both fluorescent and luminescent assays with no spectral filter requirements. It allows straightforward normalization studies and conservation of precious samples and reagents. It offers valuable new applications that should be advantageous for the drug discovery process.

## Supplementary Material

Supplemental data

## Abbreviations Used

ATP: adenosine triphosphate
DMSO: dimethyl sulfoxide
DSS: drug sensitivity score
EC_50_: half-maximal effective concentration
GCM: gas control module
ND: not determined
RLU: relative light unit
S/B: signal-to-background
sDSS: selective drug sensitivity score
S/N: signal-to-noise
