# Supplementary material for: Bioluminescent, Nonlytic, Real-Time Cell Viability Assay and Use in Inhibitor Screening
Source: Assay Drug Dev Technol. 2015 Oct 1;13(8):456–65. doi: 10.1089/adt.2015.669 (PMC4605357; doi:10.1089/adt.2015.669)
Supplement: Supplemental data [file Supp_Data.docx]

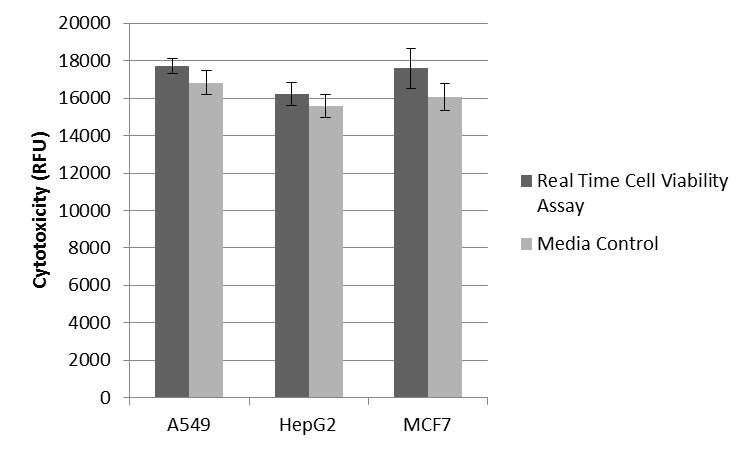


Supplementary figure 1

**Supplementary Figure 1: Real time cell viability reagents are well-tolerated by cells**. A549 cells (1000/well), HepG2 cells (500/well), and MCF7 cells (500/well) were plated in a 384-well black plate. Either media only or media containing 2x RealTime-Glo MT Cell Viability Assay reagents were added to the cells in an equal volume. The assay plate was incubated in a cell culture incubator (37^o^C/5% CO_2_) for 72 h. After 72 h, media was removed from the wells and combined with an equal volume of 2x CytoTox-Fluor Cytotoxicity Assay reagent (Promega, Madison, WI) in a new plate. The reaction was incubated in a cell culture incubator (37^o^C/5% CO_2_) for 2 hours and fluorescence was read on a Tecan M1000 Pro (excitation 485 nm, emission 520 nm, bandwidths 10 nm, optimal gain).

a


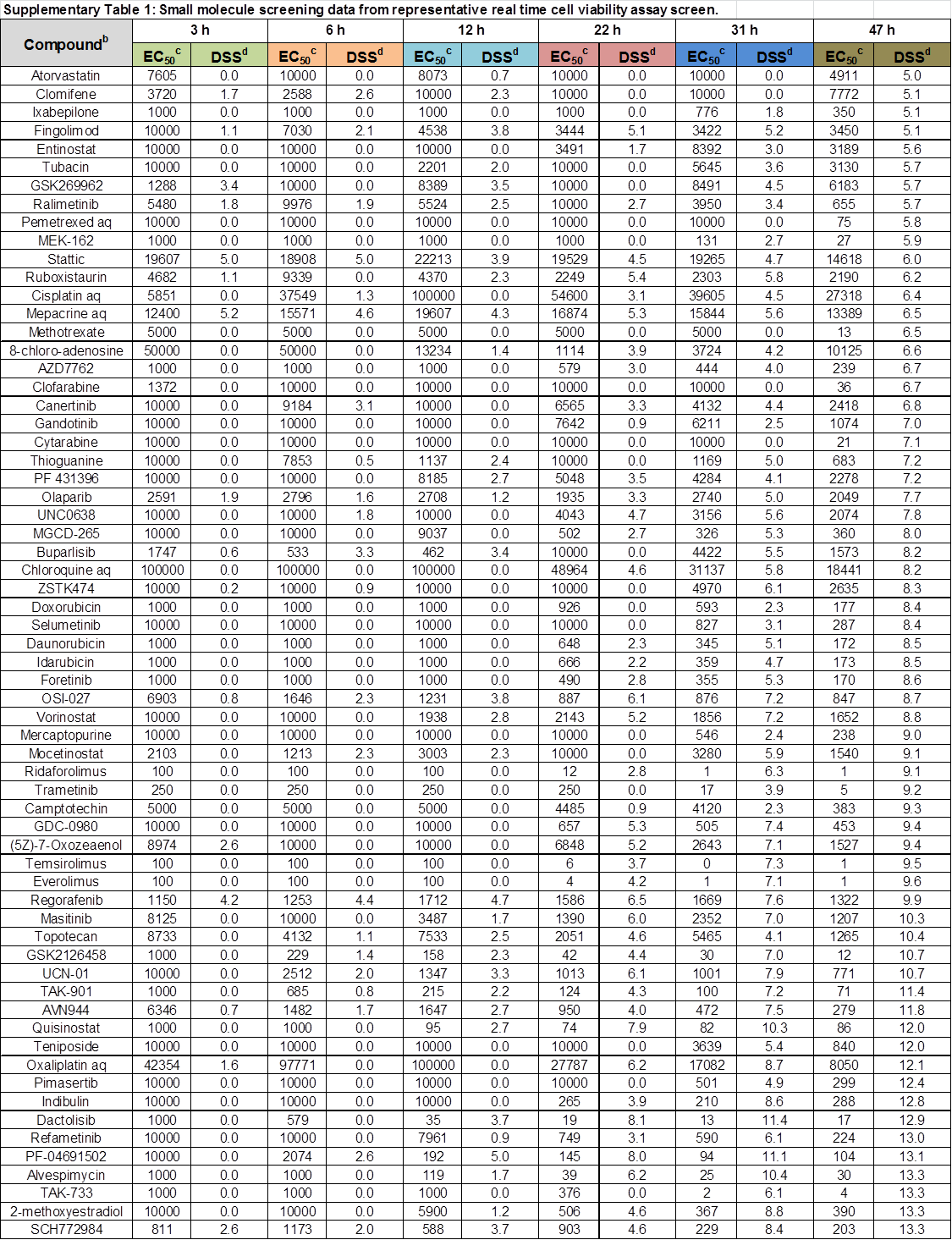


^a^The data are from one representative screen performed with the real time cell viability assay.

^b^The table contains all compounds with DSS ≥ 5 at the 47 hour reading.

^c^EC50, Half-maximal effective concentration, nM

^d^DSS, Drug Sensitivity Score

^a^The data are from one representative screen performed with the real time cell viability assay.

^b^The table contains all compounds with DSS ≥ 5 at the 47 hour reading.

^c^EC_50_, Half-maximal effective concentration, nM

^d^DSS, Drug Sensitivity Score

Supplementary Table 2: Small molecule screening data from the ATP level cell viability assay.

^a^The table contains all compounds with DSS ≥ 5.

^b^EC_50_, Half-maximal effective concentration, nM

^c^DSS, Drug Sensitivity Score

Supplementary Table 3: Small molecule screening data from the live cell protease cell viability assay.

^a^The table contains all compounds with DSS ≥ 5.

^b^EC_50_, Half-maximal effective concentration, nM

^c^DSS, Drug Sensitivity Score
